# Influence of bone density on implant stability parameters and implant success: a retrospective clinical study

**DOI:** 10.1186/1472-6831-8-32

**Published:** 2008-11-24

**Authors:** Ilser Turkyilmaz, Edwin A McGlumphy

**Affiliations:** 1 Department of Prosthodontics, Dental School at San Antonio, University of Texas Health Science Center, San Antonio, Texas, USA; 2 Department of Restorative and Prosthetic Dentistry, College of Dentistry, the Ohio State University, Columbus, Ohio, USA

## Abstract

**Background:**

The aim of the present clinical study was to determine the local bone density in dental implant recipient sites using computerized tomography (CT) and to investigate the influence of local bone density on implant stability parameters and implant success.

**Methods:**

A total of 300 implants were placed in 111 patients between 2003 and 2005. The bone density in each implant recipient site was determined using CT. Insertion torque and resonance frequency analysis were used as implant stability parameters. The peak insertion torque values were recorded with OsseoCare machine. The resonance frequency analysis measurements were performed with Osstell instrument immediately after implant placement, 6, and 12 months later.

**Results:**

Of 300 implants placed, 20 were lost, meaning a survival rate of %. 93.3 after three years (average 3.7 ± 0.7 years). The mean bone density, insertion torque and RFA recordings of all 300 implants were 620 ± 251 HU, 36.1 ± 8 Ncm, and 65.7 ± 9 ISQ at implant placement respectively; which indicated statistically significant correlations between bone density and insertion torque values (p < 0.001), bone density and ISQ values (p < 0.001), and insertion torque and ISQ values (p < 0.001). The mean bone density, insertion torque and RFA values were 645 ± 240 HU, 37.2 ± 7 Ncm, and 67.1 ± 7 ISQ for 280 successful implants at implant placement, while corresponding values were 267 ± 47 HU, 21.8 ± 4 Ncm, and 46.5 ± 4 ISQ for 20 failed implants; which indicated statistically significant differences for each parameter (p < 0.001).

**Conclusion:**

CT is a useful tool to determine the bone density in the implant recipient sites, and the local bone density has a prevailing influence on primary implant stability, which is an important determinant for implant success.

## Background

The use of dental implants to restore missing teeth has become increasingly widespread over the past two decades. Numerous clinical studies with dental implants have revealed encouraging outcomes [1-4]. The successful outcome of any implant procedure requires a series of patient-related and procedure-dependent parameters [5]. The volume of bone available and quality of the bone are highly associated with the type of surgical procedure and the type of implant, and both of these factors play a vital role in the success of dental implant surgery [6].

Clinical reports suggest that dental implants for the mandible have higher survival rates than those for the maxilla, especially for the posterior maxilla [7,8]. Compared to the mandible, the lower survival rate of maxillary implants loaded immediately/early after placement has also been reported [9]. Clinicians generally consider that the basic cause of the difference in the survival rates between maxilla and mandible is bone quality. Higher failure seems to be associated with the implants in which the surgeon observes a poor degree of bone mineralization or limited bone resistance by tactile assessment while drilling. It is typical that the bone around the implant has better quantity and quality in the mandible than the maxilla [10]. Because mechanical behavior of bone seems to be a vital factor in the achievement of osseointegration, several classification systems and procedures were suggested for assessing bone quality [11-15]. The most popular current method of bone quality assessment is that developed by Lekholm and Zarb, who introduced a scale of 1–4, based on both the radiographic assessment, and the sensation of resistance experienced by the surgeon when preparing the implant site [11]. The grading refers to individual experience, and furthermore, it provides only a rough mean value of the entire jaw. Therefore, their classification has recently been questioned due to poor objectivity and reproducibility [16,17]. Johansson and Strid described a technique whereby bone quality as a function of density and hardness could be derived from the torque forces needed during implant insertion [12]. They postulated that the energy used in tapping the site, before or during implant placement, is a combination of the thread placement force from the tip of the instrument and the friction created as the remaining part of a tap or implant enters the site. It has been demonstrated in ex vivo human preparations that the cutting resistance during implant installation correlates well with the bone density as assessed by microradiography [13]. These methods may provide helpful information about the bone density, but it is retrospective to patient assessment and its value to both clinician and patient is questioned as osteotomies have already been performed or implants have already been screwed. Therefore, computerized tomography (CT), which is more objective and reliable, could offer the best radiographic method for the morphological and qualitative analysis of the residual bone, and this imaging technique has been used in several studies [5,18-21]. The Hounsfield Units determined by the software programs in the CT machines ranges from -1000 (air) to 3000 (enamel). The density of structures within the image is absolute and quantitative and can be used to differentiate tissues in the region (i.e., muscle, 35–70 HU; fibrous tissue, 60–90 HU, cartilage, 80–130 HU; bone 150–1800 HU) and characterize bone quality (D1 bone, >1250 HU; D2 bone, 850–1250 HU; D3 bone, 350–850 HU; D4 bone 150–350 HU, D5 bone, <150 HU) [14]. CT enables the evaluation of proposed implant sites and provides diagnostic information that other imaging methods could not [22].

Several factors, such as implant geometry, preparation technique, and quality and quantity of local bone influence primary stability, and primary implant stability is one of the main factors influencing implant survival rates [23]. It is a prerequisite to establish mechanical rest, which seems to be essential for undisturbed healing and osseointegration [23,24]. Implant stability can be measured by non-invasive clinical test methods (i.e., insertion torque, the periotest, resonance frequency analysis) [23]. One of these quantitative methods is the insertion torque described by Johansson and Strid [12]. This method records the torque required to place the implant and provides valuable information about local bone quality. Another method, named Periotest, has been developed to measure the degree of the periodontal integration of teeth and the stiffness of the bone/implant interface [25]. The Periotest instrument measures the deflection/deceleration of a tooth or implant that has been struck by a small pistil from inside the instrument's hand piece. The contact time of the accelerated pistil to the implant, which moves according to the strike, is calculated into a value called the Periotest value [25]. However, Periotest values include only a narrow range over the scale of the instrument and thus, provide relatively less sensitive information about implant stability [25]. Therefore, its benefit on detection of osseointegration is a matter of debate. Another method, which is resonance frequency analysis with the Osstell instrument, has been introduced by Meredith and coworkers and used in clinical studies [26-28]. In resonance frequency analysis (RFA), the stiffness of the bone/implant interface is calculated from a resonance frequency as a reaction to oscillations exerted onto the implant/bone system. The implant is excited with an oscillating transducer screwed onto the implant and the resonance specific to the resonance system 'implant/bone' is captured electronically over a range of 5 to 15 kHz. The implant's own oscillation under a given transducer frequency is mainly dependent on the character of the implant's bony fixation. The unit of measurement in this approach is the implant stability quotient (ISQ) that is calculated from the resonance frequency and ranges with increasing stiffness of the interface from 0 to 100 units [26].

The purpose of the present study was to examine the correlations between the local bone density from CT, and the implant stability parameters including insertion torque and resonance frequency analysis and the implant survival rates.

## Methods

### Patients and implants

A total of 111 patient files were analysed. The mean age of the patients (55 females, 56 males) was 55 ± 11. All patients have been provided with a total of 300 implants in two clinics from 2003 to 2005. Details of the diameter and length of implants are presented in Table 1. The total of 300 implant sites consisted of 100 anterior mandibular sites, 60 posterior mandibular sites, 70 anterior maxillary sites, and 70 posterior maxillary sites. The patients enrolled in the study provided the following inclusion criteria: a) absence of uncontrolled medical conditions such as diabetes b) availability for follow-up visits. Exclusion criteria were: a) uncontrolled diabetes; b) radiation to head and neck; c) need to bone graft for the implant recipient site due to inadequate bone volume for regular platform implants. All patients have completed a 3-year follow up period.

**Table 1:** Dimension and number of implants placed and failed

Dimensions of implants(mm)	Numbers of implants placed	Number of implants failed
3.75 × 15	69	0
3.75 × 13	60	0
3.75 × 11.5	53	2
3.75 × 10	50	6
3.75 × 8.5	3	2
4 × 11.5	30	0
4 × 10	25	5
4 × 8.5 mm	10	5
		
Female	145	8
Male	155	12

The presurgical evaluation consisted of clinical and radiographic examinations including computerized tomography scans. All patients were thoroughly informed about the procedure and signed a written consent. Also, local ethic approval was obtained for the main study.

### Computerized tomography scans

**Figure 1:**
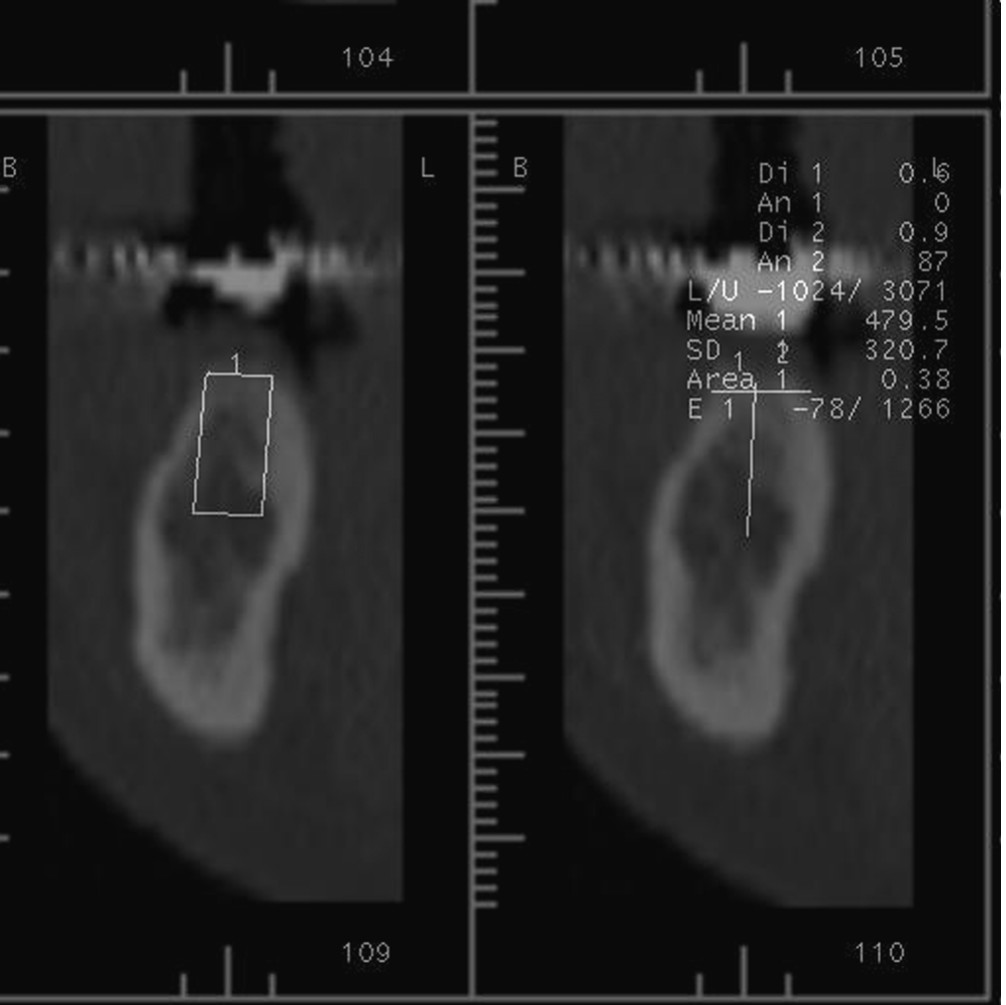
Cross-sectional CT image of the implant recipient site evaluated in this study.

In order to assess bone density of implant recipient sites, a spiral computerized tomography (CT) machine (Siemens Somatom AR-SP 40, Erlanger, Germany) was utilized. Prior to CT scan, previously fabricated surgical acrylic templates including 1 mm-diamater indicator metal rods, which were located in the center of the missing teeth, or the existing removable complete dentures attached with the same indicator rods for edentulous patients were placed in the mouth. The same scanning conditions (tube voltage 130 kV, tube current 83 mA, slice thickness 1 mm, and slice intervals 1 mm) were provided for each CT scan. The cross-sectional, coronal and axial images for each maxilla/mandible were obtained from the CT machine. The suitable implant for each previously designated implant recipient site was selected by using the cross-sectional images. The rectangular area of each implant selected was plotted on the cross-sectional images with a tool incorporated in the CT machine^15^, and the mean bone density of the implant recipient area was measured using software (Siemens Somaris/4, Erlanger, Germany) incorporated in the CT machine (Figure 1). The bone density measurements were recorded in Hounsfield units (HU).

### Surgical and prosthodontic procedures

One hour prior to implant surgery, the patients were given 2 grams of amoxicillin. Standard one-stage surgical technique was utilized to prepare the surgical sites. Full-thickness mucoperiosteal flaps were raised while the patients were under local anesthesia. 300 Brånemark Mk III TiUnite implants (NobelBiocare AB, Göteborg, Sweden) were placed under sterile saline irrigation. All drilling and implant insertion procedures were carried out with the Osseocare motor (NobelBiocare AB, Göteborg, Sweden). Immediately following implant placement, resonance frequency analysis meaurements with an Osstell instrument (Integration Diagnostics AB, Göteborg, Sweden) were performed.

Conventional (3 months or 6 months after implant placement) and early loading (1-week, 6-week, and 8-week after implant placement) protocols were used for the implants. The prostheses delivered to the patients were comprised of single-implant crowns, implant-supported fixed partial/full prostheses, and implant-supported overdentures.

### Insertion torque measurements

During the implant insertion, the maximum insertion torque value was recorded by means of the same OsseoCare motor (NobelBiocare AB, Göteborg, Sweden). Starting from 20 Ncm, the placement torque was increased in steps of 5 Ncm, when the rotation stopped because of friction before the implant was fully inserted. The OsseoCare motor was developed to provide a well-controlled insertion torque to avoid mechanical overload of the equipment or bone tissue. The final maximum insertion torque value of each implant was recorded in 20, 32, and 45 Ncm.

### Resonance frequency analysis

The resonance frequency analysis (RFA) measurements were performed using the Osstell instrument (Integration Diagnostics AB, Göteborg, Sweden). All RFA measurements were performed at implant level immediately after implant placement, and at 6- and 12-month follow-up visits. Therefore, the prostheses and abutments were removed in order to perform RFA measurements. In essence, the 8.5 mm-height transducer was mounted on the implants orthoradially with the upright part on the oral side. The resonance frequency analysis transducer was designed as an offset cantilever beam with attached two piezoceramic elements. Exciting these elements vibrates the beam. The excitation signal is a sine wave typically varying in frequency from 5 to 15 Hz with a peak amplitude of 1 V. The captured data (RF values) are recorded in Implant Stability Quotient (ISQ) ranging from 1 to 100. ISQ values are derived from the stiffness (N/μm) of the implant/bone system and the calibration parameters of the transducer. High ISQ value indicates high stability, whereas low value indicates a low implant stability.

### Implant success and failure criteria

Implants had to meet the following criteria, which are a modification of the proposal by Albrektsson and Zarb [29], to be regarded as successful: (1) no radiolucent zone around the implant; (2) the implant is acting as an anchor for the functional prosthesis; (3) confirmed individual implant stability; and (4) no suppuration, pain, or ongoing pathologic processes. All implants that failed to fulfill these success criteria were regarded as failures. Only the implants failed before prosthesis delivery were considered for this study in order to disregard the effect of various loading procedures on implant success.

### Statistical analysis

SPSS statistical software (SPSS Inc., Chicago, IL, USA) was used for all statistical analysis. The distribution of data was non-parametric, which was determined by the Kolmogorov-Smirnov test. Mann Whitney U test was used to verify possible differences between groups in terms of the bone density, insertion torque, and resonance frequency values. Correlations between the bone density, insertion torque, and implant stability values were determined by using Spearman's rho test. *P *< 0.05 was considered statistically significant.

## Results

One hundred and eleven patients (55 females, 56 males, mean age 55 ± 11) receiving 300 dental implants were included in this study. Twenty implants were lost in 15 patients, resulting in a failure rate of 6.7% after three years (average 3.7 ± 0.7 years). Only the implants failed before prosthesis delivery were included in the present study and no implant was lost after prosthesis delivery. The distribution of the failed implants was presented in Table 1.

The total of 300 implant sites consisted of 100 anterior mandibular sites (846 ± 234 HU), 60 posterior mandibular sites (526 ± 107 HU), 70 anterior maxillary sites (591 ± 176 HU), and 70 posterior maxillary sites (403 ± 95 HU). It was found that the bone density in all patients ranged from 199 HU to 1231 HU. The mean bone density, insertion torque and RFA recordings of all 300 implants were 620 ± 251 HU, 36.1 ± 8 Ncm, and 65.7 ± 9 ISQ at implant placement respectively; which indicated statistically significant correlations between bone density and insertion torque values (r = 0.768, p < 0.001), bone density and ISQ values (r = 0.882, p < 0.001), and insertion torque and ISQ values (r = 0.764, p < 0.001).

**Figure 2:**
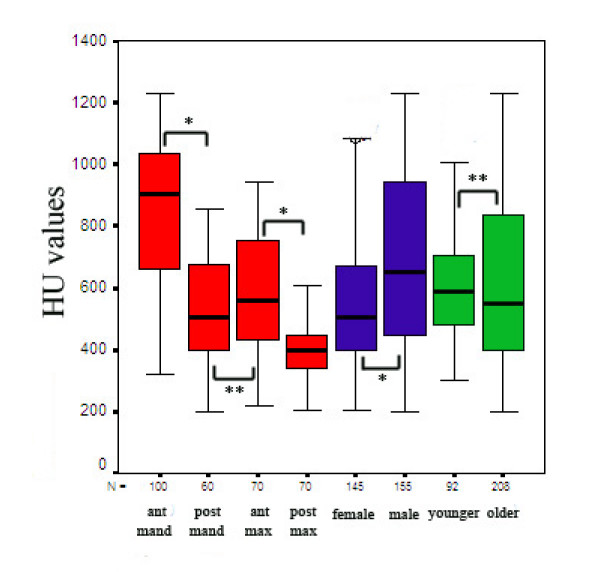
The changes in ISQ values according to the implant positions at implant surgery, 6- and 12 month follow-up recalls (N = number of implants; *p < 0.05; **p < 0.001; ***p > 0.05).

The mean bone density, insertion torque and RFA values were 645 ± 240 HU, 37.2 ± 7 Ncm, and 67.1 ± 7 ISQ for 280 successful implants at implant placement, while corresponding values were 267 ± 47 HU, 21.8 ± 4 Ncm, and 46.5 ± 4 ISQ for 20 failed implants, and the differences between succesful and failed implants were statistically significant for each parameter (p < 0.001). When 280 successful implants were considered, the mean ISQ values slightly decrease from implant surgery (67.1 ± 7 ISQ) to 6- month follow-up visit (66.9 ± 6 ISQ) (p > 0.05), and increased from 6-month to 12-month follow-up visit (68.6 ± 7 ISQ) (p < 0.001). The detailed analysis of the changes in ISQ values during the 1-year observation period and statistical analysis were presented in Figure 2.

**Figure 3:**
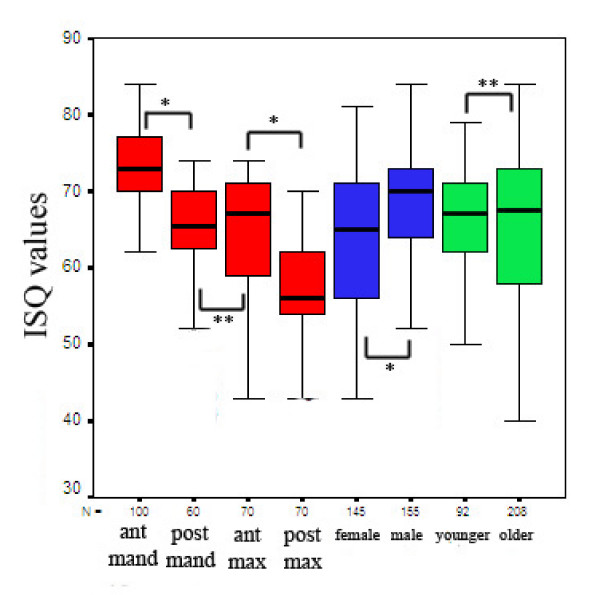
Mean bone densities according to the implant positions, gender and age of the patients (N = number of implants; *p < 0.001; **p > 0.05).

**Figure 4:**
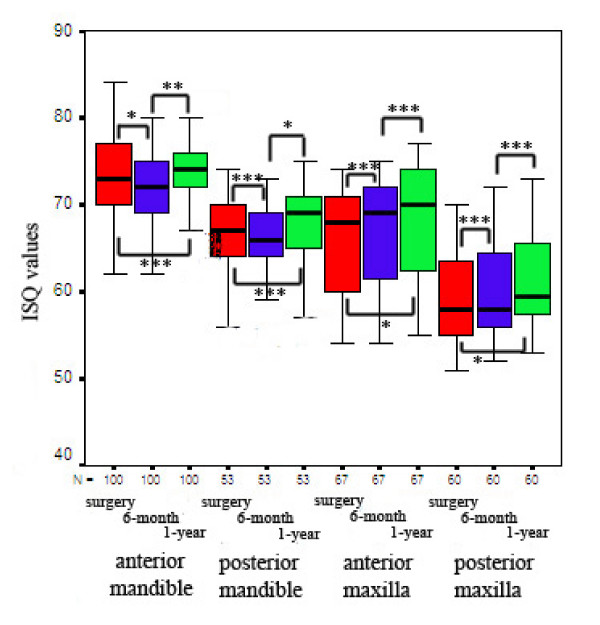
ISQ values according to the implant positions, gender and age of the patients (N = number of implants; *p < 0.001; **p > 0.05).

The mean bone density, insertion torque and RFA values were 542 ± 20 HU, 34.5 ± 8 Ncm, and 64 ± 9 ISQ for 145 implants placed in females, while corresponding values were 692 ± 271 HU, 37.6 ± 8 Ncm, and 67.3 ± 8 ISQ for 155 implants placed in males, and the differences between females and males were statistically significant for each parameter (p < 0.001) (Figures 3, 4).

## Discussion

The bone density recordings in the present study were 846 ± 234 HU, 526 ± 107 HU, 591 ± 176 HU, 403 ± 95 HU in the anterior mandible, posterior mandible, anterior maxilla, and posterior maxilla respectively, which are comparable with those in the previous reports [17,18]. The study regarding 139 implant recipient areas by Norton and Gamble disclosed that the mean bone densities were 970 HU, 669 HU, 696 HU, and 417 HU in the anterior mandible, the posterior mandible, the anterior maxilla, and the posterior maxilla respectively [18]. Another previous study with 219 implant sites by Shapurian et al., included that the mean bone density recordings in the anterior mandible, the anterior maxilla, the posterior maxilla, the posterior mandible were 559 HU, 517 HU, 333 HU, and 321 HU [17]. The differences between the present and previous studies might come from the distribution of implant recipient sites and the variations in the age and gender of patients. Also, higher mean bone density value of the implant sites was found in males than females in the present study, and this finding may be related to the hormonal peculiarities in females and generally higher bone mass in males. An earlier study including the measurement of the bone mineral contents in the jaws and forearms have disclosed lower bone mineral densities in females in comparison with males and larger bone mineral content loss in elderly females throughout adult life [20].

The strong correlations between bone density and insertion torque values, bone density and ISQ values, and insertion torque and ISQ values in the present study are consistent with a previous study by Turkyilmaz and coworkers [19]. This clinical study with 158 implant sites from 85 patients indicated strong correlations between bone density values from CT and stability parameters. These findings are partially in agreement with the earlier studies [30,31]. Friberg et al., compared cutting torque and resonance frequency measurements of TiUnite MK II implants placed in the maxilla and found a significant correlation, only in the crestal third of the implants, between placement torque and resonance frequency at implant placement [30]. Da Cunha et al., determined significant linear correlations between the placement torques for apical, middle, and crestal third, and resonance frequency analysis values for 12 TiUnite implants placed in the maxilla [31].

The ISQ findings observed in the present study can be compared with those in the previous studies [32-35]. Cornelini et al., placed 40 implants in twenty patients with missing mandibular premolars and molars [32]. For 39 successful implants as one implant was lost, the mean ISQ values were 72 and 74.5 at implant surgery and after one year, which was not statistically significant. The follow-up study by Degidi et al., included 802 dental implants placed in 321 patients, and minimum observation period was one year for each implant in that study [33]. In that study, the failed implants showed a mean ISQ value of 46, while the successfully osseointegrated implants had ISQ values around 60. Glauser and coworkers inserted 81 implants in 23 patients for immediate/early loading [34]. At 1-month follow-up recall, RFA values were significantly higher for the successful implants in comparison with the failing ones. RFA also showed different patterns for failing and successful implants, and RFA values constantly decreased after implant placement for failing implants, while corresponding values for successful ones slightly decrease after implant placement and then remained stable or increased. Sjostrom et al. [35], placed 192 implants after 6 months of bone-graft healing. Implant stability was measured four times using RFA for 190 implants, and they lost 20 implants, which means a survival rate of 90% during the 3-year follow up. The implant stability quotient (ISQ) value for all implants differed significantly between abutment connection (60.2 ± 7.3) and after 6 months of bridge loading (62.5 ± 5.5) but were nonsignificant between implant placement (61.9 ± 9.5) and abutment connection (60.2 ± 7.3), and also 6 months of bridge loading (62.5 ± 5.5) and 3 years of bridge loading (61.8 ± 5.5). When comparing individual implants, the mean ISQ at placement for 170 successful implants was 62.6 ± 11.1 compared to 54.9 ± 11.1 for 20 failed implants, which indicated a significant difference. In the present study, when compared to the failed implants, the higher ISQ values were found in the successful implants, and when all successful implants were considered the ISQ values slightly decreased following implant placement and then increased up to 1-year.

In the present study, 121 implants were placed in smokers and 11 implants were lost (9.1%) while 179 implants were placed in non-smokers and 9 implants were lost (5.02%). When compared to the non-smokers, the higher percentage of implant failures in the smokers observed in the present study is in agreement with the earlier studies [36,37]. Strietzel et al., revealed significantly enhanced risks of biologic complications among smokers and concluded that smoking is a significant risk factor for dental implant therapy and augmentation procedures accompanying implantations [36].

As an alternative to CT scanning, laser Doppler flowmetry has recently been introduced as a valid method of determining bone vascularity and, as a derivation, bone quality [38]. Verdonck et al., extracted all maxillary and mandibular premolars and molars of six minipigs [38]. The maxilla and mandible of three minipigs received three irradiation exposures at a total dose of 24 Gy. After irradiation, five initial implant holes were drilled in the residual alveolar ridge of each edentulous site. In order to assess bone vascularity, laser Doppler flowmetry recordings were carried out in the initial holes. A total of 120 implants were placed in the six minipigs. Subsequently, and at 8, 16, and 24 weeks after implant placement, implant stability was recorded by resonance frequency analysis. They concluded that laser Doppler flowmetry is an adequate, reproducible, and reliable method for assessing alveolar bone vascularity.

## Conclusion

Under the guidelines of this study, the following conclusions can be drawn:

1. CT scanning, which is a non-invasive method may be used to determine the regional bone quality before implant surgery.

2. Significant correlations found between bone quality and implant stability parameters indicate that clinicians may predict primary stability before implant insertion, and they may modify their treatment plans (i.e., implant locations, longer healing periods) before implant surgery, where the bone quality is poor.

## Authors Contributions

IT  gathered and analyzed all retrospective data, and did statistics. Also wrote the main version of the article.     EG wrote the final version of the article and did corrections.   

## Competing interests

The authors declare that they have no competing interests.

## Pre-publication history

The pre-publication history for this paper can be accessed here:


